# Hypofucosylation of Unc5b regulated by Fut8 enhances macrophage emigration and prevents atherosclerosis

**DOI:** 10.1186/s13578-023-00959-y

**Published:** 2023-01-20

**Authors:** Xi Yang, Limei Ma, Jun Zhang, Linmu Chen, Zhen Zou, Di Shen, Hui He, Lei Zhang, Jun Chen, Zhiyi Yuan, Xia Qin, Chao Yu

**Affiliations:** 1 Institute of Life Sciences, Chongqing Medical University, Chongqing, 400016 China; 2 College of Basic Medicine, Inner Mongolia Medical University, Hohhot, 010110 China; 3 College of Pharmacy, Chongqing Medical University, Chongqing, 400016 China

**Keywords:** Unc5b, Fut8, Fucosylation, Macrophages, Atherosclerosis

## Abstract

**Background:**

Atherosclerosis (AS) is the leading underlying cause of the majority of clinical cardiovascular events. Retention of foamy macrophages in plaques is the main factor initiating and promoting the atherosclerotic process. Our previous work showed that ox-LDL induced macrophage retention in plaques and that the guidance receptor Uncoordinated-5 homolog B (Unc5b) was involved in this process. However, little is known about the role of Unc5b in regulating macrophage accumulation within plaques.

**Results:**

In the present study, we found that Unc5b controls macrophage migration and thus promotes plaque progression in ApoE^−/−^ mice. The immunofluorescence colocalization assay results first suggested that fucosyltransferase 8 (Fut8) might participate in the exacerbation of atherosclerosis. Animals with Unc5b overexpression showed elevated levels of Fut8 and numbers of macrophages and an increased lesion size and intimal thickness. However, these effects were reversed in ApoE^−/−^ mice with Unc5b knockdown. Furthermore, Raw264.7 macrophages with siRNA-mediated silencing of Unc5b or overexpression of Unc5b were used to confirm the regulatory mechanisms of Unc5b and Fut8 in vitro. In response to ox-LDL exposure, Unc5b and Fut8 were both upregulated, and macrophages showed reduced pseudopod formation and migratory capacities. However, these capacities were restored by blocking Unc5b or Fut8. Furthermore, the IP assay indicated that Fut8 regulated the level of α-1,6 fucosylation of Unc5b, which mainly occurs in the endoplasmic reticulum (ER), and genetic deletion of the main fucosylation sites or Fut8 resulted in hypofucosylation of Unc5b. Moreover, the macrophage migration mediated by Unc5b depended on inactivation of the p-CDC42/p-PAK pathway. Conversely, macrophages with Unc5b overexpression displayed activation of the p-CDC42/p-PAK pathway and decreased migration both in vivo and in vitro.

**Conclusion:**

These results demonstrated that hypofucosylation of Unc5b regulated by Fut8 is positively associated with the delay of the atherosclerotic process by promoting the migration of foamy macrophages. These findings identify a promising therapeutic target for atherosclerosis.

**Supplementary Information:**

The online version contains supplementary material available at 10.1186/s13578-023-00959-y.

## Introduction

Atherosclerosis (AS) continues to be a common chronic inflammatory vascular disorder that is responsible for deaths related to cardiovascular disease (CVD) worldwide [1–4]. Plaques containing various inflammatory cells and necrotic cells that form around the arterial wall are considered the typical pathology of AS [5]. Macrophages, especially those that engulf cholesterol (foam cells), are recognized as the fundamental mediators of plaque initiation and development [6, 7]. It has been reported that macrophages can emigrate at the early stage of plaque formation during atherosclerosis regression, but they become foam cells and gradually lose the capacity for emigration during the progression of atherosclerotic lesions. Therefore, the imbalance between retention and migration causes more macrophages to accumulate in atherosclerotic plaques [5–9]. Although the accumulation of macrophages has long been considered a major inducer of AS progression, research on the mechanism that regulates the migration and retention of foamy macrophages within plaques is largely incomplete [10].

Netrin-1 is a laminin-like matrix protein that belongs to the axonal guidance protein family. Researchers have found that netrin-1 is essential for inhibiting the migration of monocytes, neutrophils, and lymphocytes [11, 12]. Recently, researchers further confirmed that netrin-1 functions as a principal indicator of the macrophage foaming process by binding to its receptor Unc5b [2, 11, 13–16]. In particular, Unc5b expression is strongly promoted in foam cells, thereby leading to its interaction with netrin-1 and further inhibiting the emigration of foam cells from plaques, as verified by our previous work, indicating that Unc5b strongly contributes to macrophage migration and retention processes [11]. However, the underlying mechanism by which Unc5b controls foam cell formation and retention within plaques, as well as its corresponding downstream signals, remains poorly defined. Thus, identification of its regulatory mechanisms may lead to the discovery of novel therapeutic targets for atherosclerosis.

Glycosylation is a universal posttranslational modification that occurs in the endoplasmic reticulum (ER) and Golgi apparatus [17, 18]. More than 50% of proteins can be glycosylated by the covalent attachment of a saccharide to a polypeptide backbone via an N-linkage to asparagine (Asn) or an O-linkage to Ser/Thr, thus mediating receptor‒ligand interactions and stabilizing protein structures [19, 20]. Fucosyltransferase 8 (Fut8) belongs to the fucosyltransferase family and is the key enzyme involved in protein glycosylation, especially N-glycan core fucosylation [18, 21]. Researchers have found that aberrant glycosylation mediated by Fut8 is often involved in the decreased mobility of foam cells and is responsible for the accumulation of cholesterol-enriched foam cells in the intima in the early stage of atherosclerosis [22–25]. Unc5b is a guidance receptors and is also a highly glycosylated protein [25–28]. Whether Fut8 regulates Unc5b glycosylation and by which mechanism glycosylated Unc5b affects the Unc5b-netrin-1 interaction and further affects foam cell formation and retention remain elusive.

In the present study, we validated the key role of Unc5b in the foam cell retention process both in vitro and *in vivo.* The results showed that the level of Unc5b was strongly associated with macrophage migration and the plaque lesion size in ApoE^−/−^ mice as well as in Raw264.7 cells. Importantly, we also found that hypofucosylation of Unc5b regulated by Fut8 enhanced macrophage emigration from intimal lesions and further prevented atherosclerosis through the p-CDC42/p-PAK signaling pathway. These findings may help to identify biomarkers or develop combination treatment strategies for atherosclerosis.

## Results

### Unc5b contributes to the formation of atherosclerotic plaques

**Fig. 1:**
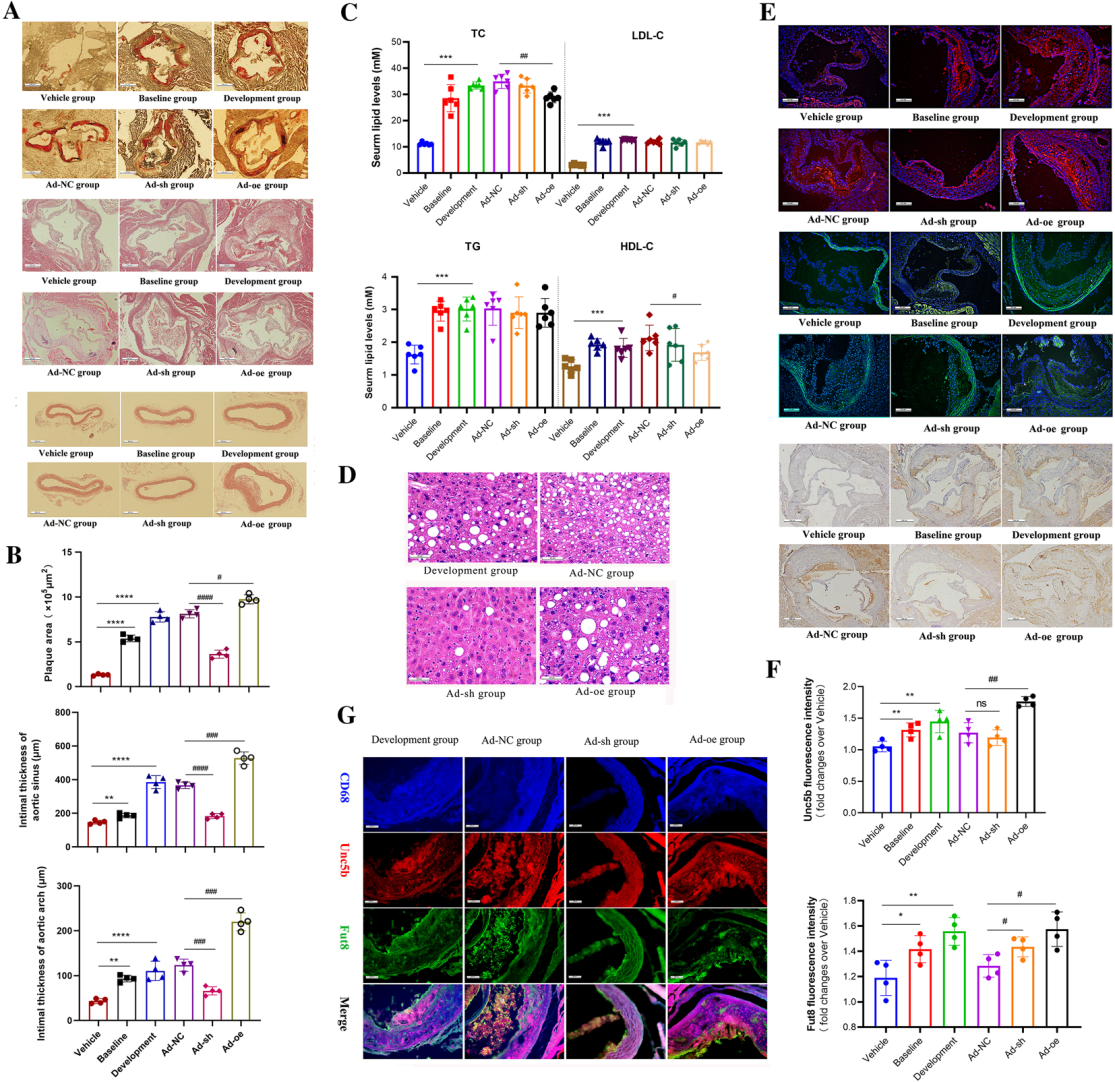
Unc5b contributes to the formation of atherosclerotic plaques. **A** Representative Oil Red O-stained images of aortic valve sections; The intimal thickness of the aortic valve is shown by H&E staining; The aortic arch was stained with H&E to reveal the intimal thickness (bar=500 μm). **B** Quantitative image analysis of Figure A. **C** Serum lipid levels such as TC, TG, LDL-C, HDL-C in different groups. **D** Livers from ApoE^−/− ^mice in different group were stained with H&E, and the number of vacuolized structure was characterized as maker to present the level of liver lipid and necrosis. **E** Atherosclerotic aortic sinus were collected from ApoE^−/−^ mice and immunostaining assay was performed on the aortic to detect the expression of Unc5b as well as Fut8 in plaque (bar=200 μm, n=4). Unc5b (red), Fut8 (green), DAPI (blue); The content of macrophage (CD68^+^) in aortic valve plaques was determined by immunohistochemistry (bar=500 μm, n=4). **F** Quantification of Unc5b and Fut8 expression from immunostaining assay. Data are reported as the mean ± S.D. **G** Atherosclerotic aortic sinus were collected from ApoE^−/−^ mice in Development group, Ad-NC, Ad-sh and Ad-oe group. The aortic sinus was stained for Unc5b (red), Fut8 (green), CD68 (blue) and their co-localization (yellow merge). Data are reported as the mean ± S.D. (n＞3, ****, p< 0.0001vs vehicle group, ###, p< 0.001 vs Ad-NC group, t-test). The mice in the Ad-NC group, Ad-sh group and Ad-oe group were given NC or Unc5bshRNA or Unc5b over-expression adenovirus injection into the caudal vein for four weeks.

To investigate whether Unc5b is involved in the development of atherosclerosis, we first established an animal model of atherosclerosis in ApoE^−/−^ mice and divided them into three groups, namely, the vehicle group, baseline group, and atherosclerosis development group, as described in the Materials and Methods section. The results showed that the food intake and body weight were not significantly different among the three groups (Additional file 1: Fig. S1A). However, the concentrations of serum lipids, including low-density lipoprotein cholesterol (LDL-C), total cholesterol (TC) and triglycerides (TGs), were higher in the atherosclerosis development group than those in the vehicle group (Fig. 1C). In addition, the weights of the liver, heart and spleen were higher in the atherosclerosis development group than in the vehicle group (Additional file 1: Fig. S1B). Moreover, we used Oil Red O staining combined with H&E staining to evaluate the lipid content in the plaque area and pathological structure. The results shown in Fig. 1 A and B indicate that the plaque area in the development group was much larger than that in the baseline group and vehicle group. Additionally, H&E staining showed that the intimal thickness of the aortic sinus and the thickness of the aortic arch were increased. Together, these results indicated that the atherosclerosis development model was successfully established (Fig. 1A, B).

Furthermore, to determine whether Unc5b regulates the atherosclerotic process, we established an animal model by injecting pHBAd-U6-CMV-Vector Unc5b knockdown adenovirus (Ad-sh) or Unc5b overexpression adenovirus (Ad-oe) into the caudal veins of development ApoE^−/−^ mice for four weeks. The mice in Ad-NC group was injected empty adenovirus and conducted as control group. Then, we analyzed the plaque area, intimal thickness and lipid levels by using Oil Red O staining, H&E staining and biochemical analysis, respectively. The results in Fig. 1A-C show that animals injected with Unc5b shRNA displayed strong antiatherosclerotic activity, exhibiting fewer advanced plaques and a lower intimal thickness compared with mice in Ad-NC group. However, animals injected with the Unc5b overexpression adenovirus exhibited more severe plaque progression and an increased intimal thickness compared with mice in Ad-NC group. Moreover, serum lipid analysis as well as liver H&E staining showed that Unc5b participates in the regulation of serum lipid levels as well as lipid accumulation during liver metabolic processes and that these effects were not completely associated with the progression of atherosclerotic lesions. Ad-sh treatment improved serum lipid levels and reduced hepatic steatosis, while Ad-oe treatment enhanced hepatocyte vacuolation and lipid accumulation compared with Ad-NC treatment, as shown in Fig. 1C–D. Together, these results indicated that Unc5b promoted the progression of atherosclerotic lesions, which was partially independent of the lipid metabolism abnormalities.

In addition, we investigated Unc5b expression in atherosclerotic plaques. The results indicated that the level of Unc5b was greatly increased with plaque progression compared with the vehicle group as shown in Fig. 1E and F. Additionally, staining of Fut8 confirmed that higher Unc5b expression was associated with higher Fut8 expression in atherosclerotic plaques. However, the expression of Fut8 in mice injected with Ad*-*sh was higher than that in mice injected with Ad-NC, which may suggest that Unc5b cannot directly affect the Fut8 level in vivo. Analysis of CD68^+^ positive cells showed fewer macrophages in the plaques of animals with lower Unc5b and Fut8 expression. Moreover, an immunofluorescence colocalization assay of Unc5b, Fut8 and CD68 was used to determine their interaction in aortic sinus. And the mice from development group as well as Ad-NC group could be identified as control group. In order to exclude the possibility of spontaneous fluorescence of antibody, we applied IgG staining which distinguished the primary antibody of CD68, Fut8 as well as Uuc5b in atherosclerotic aortic sinus. The results as shown in Additional file 2: Fig. S2 indicated that the aortic sinus staining with normal IgG have not display auto fluorescent coming from elastic laminar, which suggested that immunofluorescent staining of CD68, Fut8 as well as Unc5b in aortic sinus were specificity. Then we found that the colocalization of Unc5b, Fut8 and CD68 in the aortic sinus were strongly promoted during the development of atherosclerosis as indicated in the Development group (Fig. 1G). And these proteins were especially colocalized in the subendothelial layer of the aorta. However, mice treated with Ad-sh displayed lower Unc5b/Fut8/CD68 colocalization than those treated with Ad-oe. Interestingly, we also found that mice injected with Ad-sh displayed lower levels of CD68 compared with mice in Ad-NC group, which suggested that Unc5b can affect macrophages aggregated in the subendothelial space. Together, these results suggested that Unc5b facilitates atherosclerotic plaque progression, and may participate in the regulation of the macrophage content in plaques in cooperation with Fut8, which indicated that Unc5b may serve as a potential therapeutic target.

### Unc5b suppresses the migration of macrophage foam cells

**Fig. 2:**
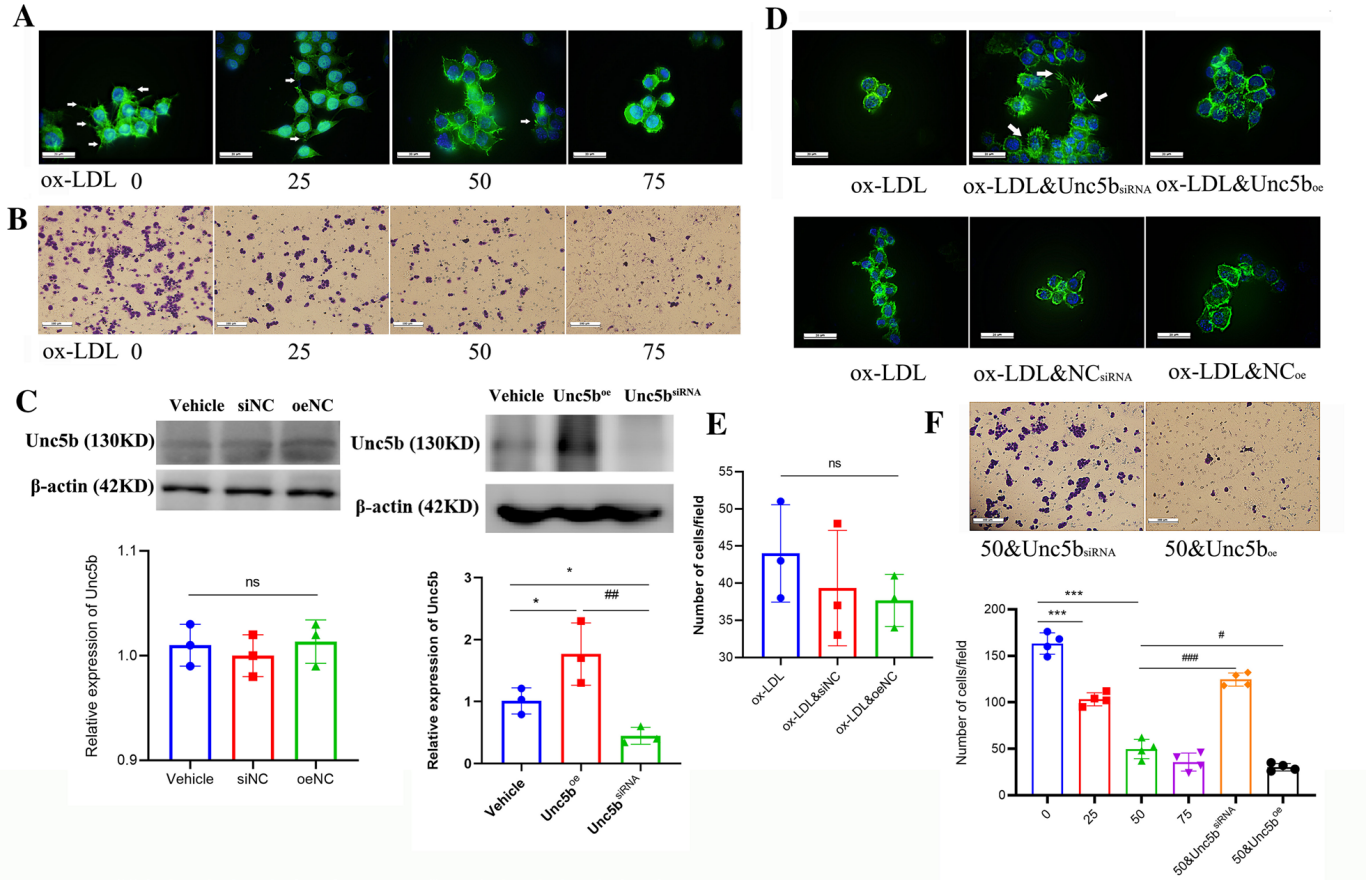
Unc5b suppresses migration of macrophage foam cells Raw 264.7 cells were subjected to various concentrations of ox-LDL (0, 24, 50 and 75 μg/ml) for 24 h, F-actin was stained with phalloidin-iFluor 488 (green), and the nuclei were counterstained with DAPI (blue), pseudopodia of macrophages were marked in white arrows (**A**), the number of cells on the dark side was identified by Transwell assay (**B**). (**C**) Western blot analysis of Unc5b protein levels in Raw 264.7 cells with Unc5b^oe^ or Unc5b^siRNA^. Quantification of Unc5b expression from Western blotting. Data are reported as the mean ± S.D. (n＞3, * p< 0.05vs vehicle group, ^##^p< 0.01 vs Unc5b^oe^ group, t-test). (**D**) Raw 264.7 macrophages were pretreated with Unc5bsiRNA or pcDNA3.1-Unc5b plasmid combined with ox-LDL for another 24 h, cells were stained by F-actin (green), DAPI (blue), and pseudopodia (marked in white). (E, F) Raw 264.7 cells were pretreated with empty plasmid or Unc5b^siRNA^ or Unc5b^oe^, the number of cells on the dark side was identified by Transwell assay upon ox-LDL (50 μg/ml) treatment. Data are reported as the mean ± S.D. (bar=100 μm, n=4, ^***^, p< 0.001 vs ox-LDL 0 μg/mL group,^###^, p< 0.001 vs ox-LDL 50 μg/mL, t-test)

To further explore the role of Unc5b in the regulation of the macrophage content, we next used ox-LDL to treat Raw264.7 cells and established a macrophage model with low migration capability according to our previous study [11]. Filamentous actin (F-actin) is regarded as a marker to evaluate changes in the cytoskeleton, especially the formation of spindle-shaped pseudopodia, which can show the migration ability of macrophages [29, 30]. In the present study, we used F-actin staining to evaluate the formation of macrophage protrusions. First, we treated Raw264.7 cells with different concentrations of ox-LDLox-LDL for 24 h and 0 µg/mL was used as control. Then we used F-actin staining and a Transwell assay to quantify the formation of protrusions as well as determine the number of migrated cells. The results showed that the formation of macrophage protrusions was decreased upon ox-LDL treatment and was especially lower after treatment with 50 or 75 µg/mL ox-LDL compared with control group (Fig. 2A). In addition, the number of migrated cells was decreased upon ox-LDL treatment (Fig. 2B, F), and collectively, these findings confirmed our previous finding that ox-LDL significantly inhibited foam cell migration [11].

Next, we used the pcDNA3.1-Unc5b plasmid or Unc5b siRNA to establish cell models with Unc5b overexpression (Unc5b^oe^) and knockout (Unc5b^siRNA^), respectively, along with ox-LDL stimulation in 50 µg/mL and ox-LDL alone was used as control group. The results shown in Fig. 2C demonstrated that the cell models with Unc5b intervention were successfully established. Furthermore, we explored the regulatory effect of Unc5b on foam cell migration. As shown in Fig. 2D and E, transfection of the empty plasmid did not affect the formation of protrusions or macrophage migration, which were inhibited by ox-LDL. However, Unc5b^siRNA^ cells displayed restoration of macrophage pseudopodia and an increased migration capacity compared with ox-LDL control group. In contrast, Unc5b^oe^ cells had a lower capacity to migrate and fewer protrusions than cells in the ox-LDL group, as shown in Fig. 2D and F. Together, these results suggested that Unc5b suppressed the migration of macrophage foam cells.

### Fut8 suppresses macrophage migration by regulating α-1,6 fucosylation levels

**Fig. 3:**
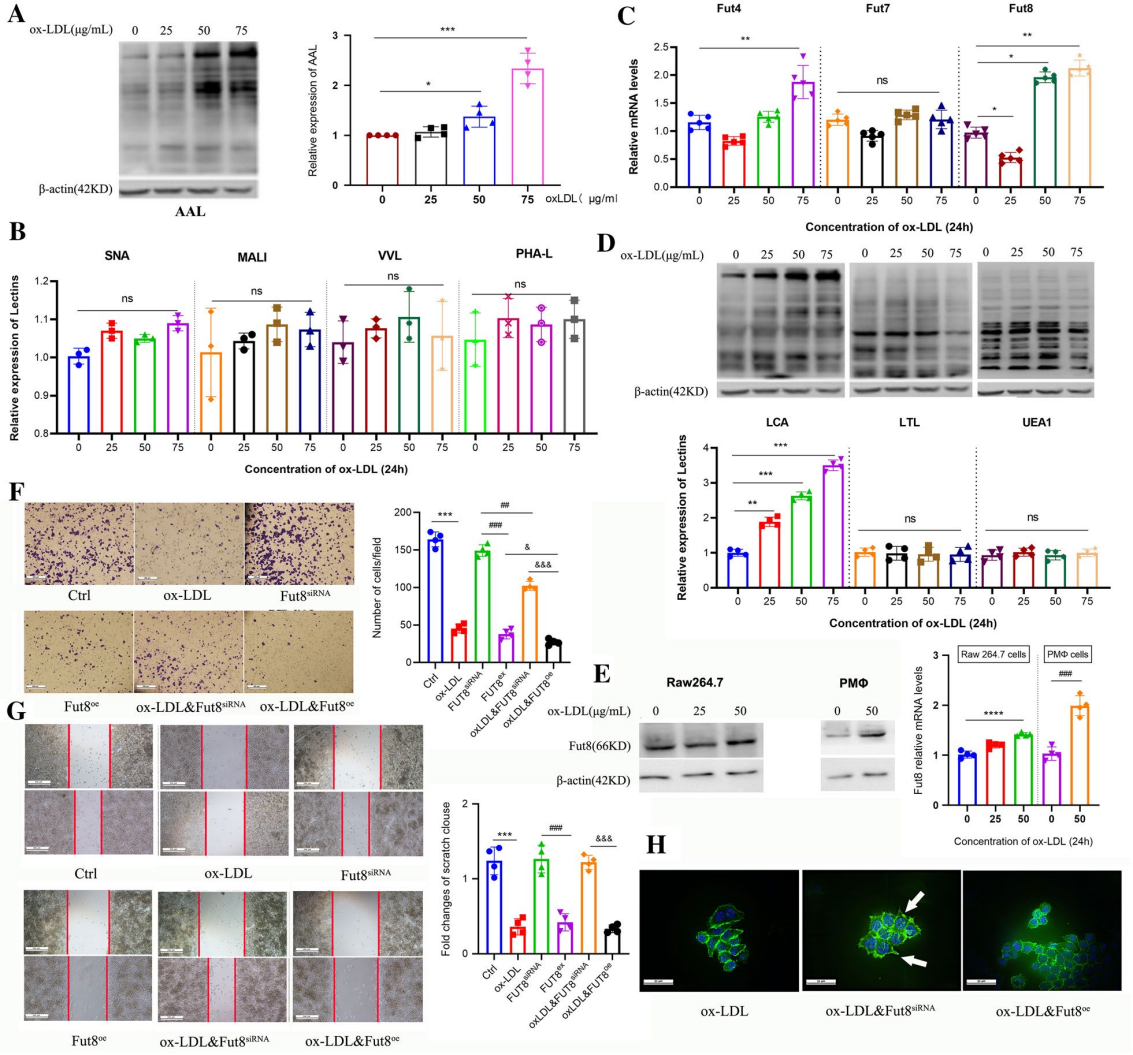
Fut8 participates in macrophage migration induced by ox-LDL. **A **The fucosylation level of the total protein of Raw 264.7 cells was determined by lectin blot after treatment with (0-75) mg/ml ox-LDL for 24 h. **B** Lectin blotting assays conducted to characterize the levels of total protein α-2,6 sialylation (SNA) and α-2,6 sialylation (MALI) levels in Raw 264.7 cells subjected to (0-75) mg/ml ox-LDL for 24 h. **C** The mRNA levels of Fut4, Fut7 and Fut8 in Raw 264.7 cells were analyzed by real-time RT-PCR after treatment with 0-75 mg/ml ox-LDL for 24 h. **D** The levels of total protein α-1,2-fucosylated (UEA1), α-1,3/4-fucosylated (LTL) and α-1,6-fucosylated (LCA) of Raw 264.7 cells were determined by lectin blot after treatment with 0-75 mg/ml ox-LDL for 24 h; **E** Western blotting was used to detect the expression of Fut8 protein in Raw 264.7 cells and mouse peritoneal macrophages after treatment with ox-LDL for 24 h. **F**,**G** Raw 264.7 cells were pretreated with siFut8 or pcDNA3.1-Fut8 plasmid, and then the number of cells on the dark side was identified by Transwell assay upon ox-LDL (50 μg/mL) treatment (bar=100 μm), the wound healing assay was used to identify the cell migration speed (bar=500 μm) or (H) the cells were stained for F-actin using Phalloidin-iFluor 488, and nuclei were counterstained with DAPI. Green:F-actin staining; Blue: DNA staining (bar=20 μm). Data were reported as the mean ± S.D. ( n=4, ^*^p< 0.05 ; ^***^p< 0.001, t-test.).

Our previous results indicated that Unc5b expression was positively correlated with the expression of Fut8, which may interact with Unc5b in macrophages in plaques. Then, we evaluated the possible involvement of Fut8 in macrophage migration induced by ox-LDL. First, we determined the total fucose glycosylation level by using fucose-specific *Aleuria aurantia* lectin (AAL). The results showed that ox-LDL strongly increased the total fucosylation level, as shown in Fig. 3A. In addition, we also determined the levels of other intracellular glycosylated forms with other lectins such as SNA (α-2,6 sialylation), MALI (α-2,3 sialylation), VVL (galactosylation), and PHA-L (galactosylation). The results showed that the expression of the above lectins in macrophages was not changed upon ox-LDL treatment (Fig. 3B), which suggested that ox-LDL mainly affects the fucosylation level in macrophages. Fut8, Fut4 and Fut7 are considered the key enzymes involved in protein fucosylation [18, 21]. We further analyzed the mRNA levels, and the results showed that the Fut8 and Fut4 mRNA levels were significantly increased upon ox-LDL treatment, whereas the Fut7 level showed no apparent change (Fig. 3C). Combined with the in vivo results, these results suggested that ox-LDL affected the fucosylation level mainly by regulating Fut8 expression in macrophages.

*Lotus tetragonolobus* lectin (LTL), *Ulex europaeus* agglutinin (UEA1) and *Lens culinaris* agglutinin (LCA) specifically binds to α-1,2 fucosylated, α-1,3/4 fucosylated and α-1,6 fucosylated glycans in cells. Therefore, we further determined which fucosylated form is involved in the process of Fut8 activation in response to ox-LDL. The results showed that ox-LDL increased the LCA level in a concentration-dependent manner but had no effect on the UEA1 and LTL levels (Fig. 3D), which suggested that ox-LDL mainly increased the level of α-1,6 fucosylation mediated by Fut8 in macrophages. To further verify that the activation of Fut8 is induced by ox-LDL, we used primary peritoneal macrophages (PMΦ) from C57BL/6J mice to confirm the results in Raw264.7 cells. As shown in Fig. 3E, the expression of Fut8 was upregulated in both cell types upon ox-LDL treatment.

Next, we performed siRNA-mediated knockout or plasmid-mediated overexpression (oe) of Fut8 in Raw264.7 cells via transfection, and cells with ox-LDL treatment alone were used as control group. After treatment with ox-LDL, we found that Fut8^oe^ cells displayed a lower migration capability and fewer migrating cells on the underside of the membrane, as shown in Fig. 3F-G. In contrast, Fut8^siRNA^ promoted more cell migration compared with that in the Fut8^oe^ group upon ox-LDL treatment. Additionally, F-actin staining showed that Fut8^oe^ macrophages displayed fewer pseudopodia, while Fut8^siRNA^ caused an increase in pseudopodia compared with ox-LDL control group, as shown in Fig. 3H. Taken together, these results indicated that Fut8 suppressed macrophage migration by regulating α-1,6 fucosylation levels in response to ox-LDL treatment.

### Hypofucosylation of Unc5b regulated by Fut8 facilitates macrophage migration

**Fig. 4:**
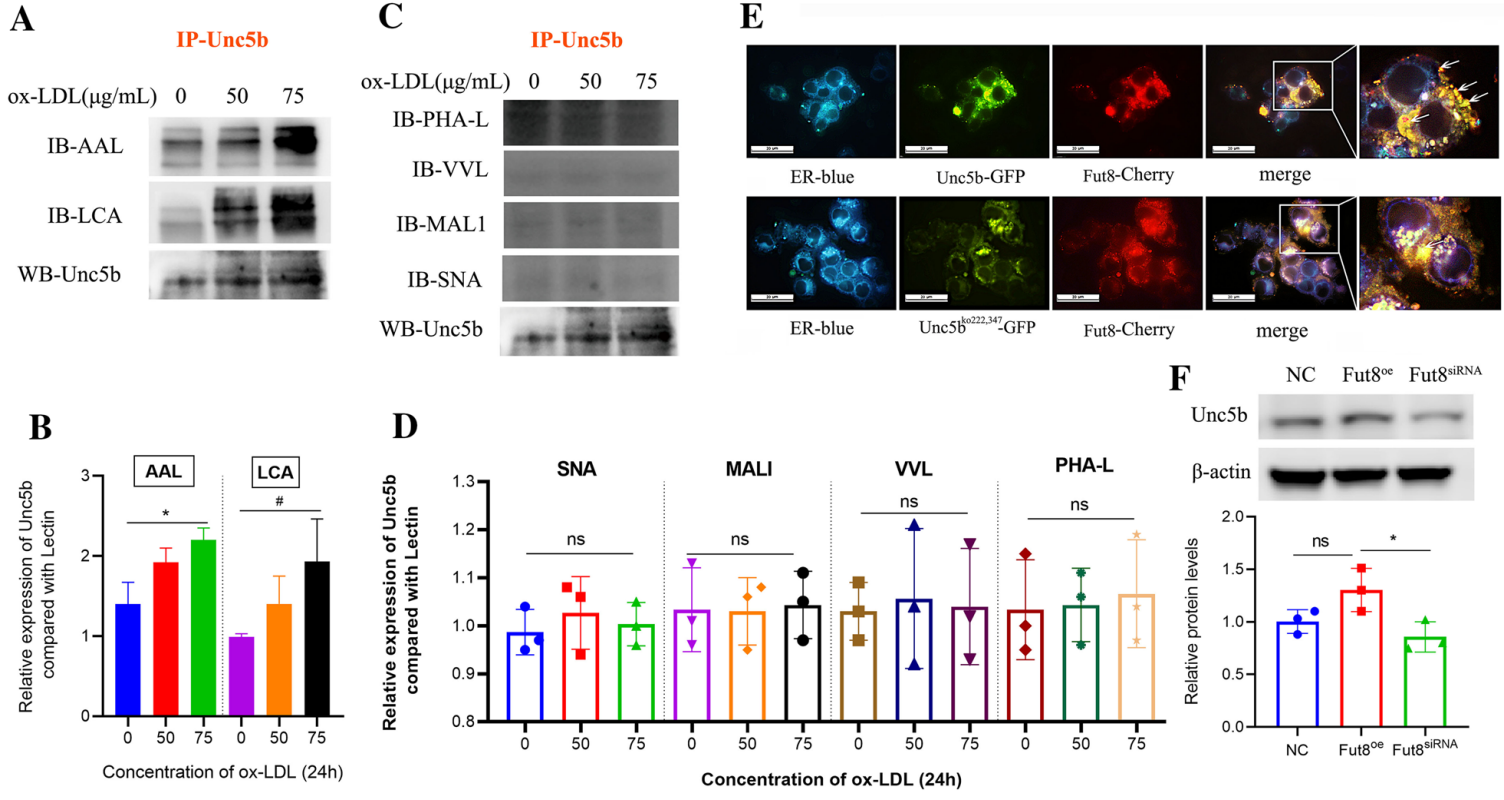
Hypofucosylation of Unc5b regulated by Fut8 facilitates macrophage migration.  **A**, **C** The interaction between Unc5b and AAL, LCA, PHAL, VVL, MAL1, SNA detected by IP assays after ox-LDL treatment for 24 h. **B**, **D** Quantification of Unc5b expression from IP assay. Data are reported as the mean ± S.D. (n＞3, t-test). **E** HEK293T cells were pretreated with pCMV-Unc5b-GFP plasmid or pCMV-Unc5bko222,347-GFP plasmid and pCMV-Fut8-mCherry plasmid and cotransfected with pCMV-Fut8-mCherry plasmid, followed by immunostaining. ER (blue), Fut8 (red), Unc5b (green), merge (yellow)(bar=20 μm, n=4). **F** The α-1,6-fucosylated protein level of Unc5b was detected by LCA in cells with Fut8eo or Fut8siRNA plasmid treatment and the quantification of protein expression was shown below the blot. Data are reported as the mean ± S.D. (n＞3, *, p< 0.05vs NC group, t-test).

Previous studies have shown that Unc5b is a single-pass transmembrane receptor protein that contains an extracellular region, a transmembrane region, and a cytoplasmic region [28, 31, 32]. Amino acid sequence analysis of Unc5b showed that there are several N-glycosylation sites in the protein sequence (https://www.uniprot.org/uniprot/q8k1s3). In particular, the extracellular region of the Unc5b receptor may undergo N-glycosylation at two sites, asparagine (ASN, n) 222 and 347. Our present in vivo study showed that Unc5b may interact with Fut8 in macrophages; thus, we further determined whether Unc5b is modified by fucosylation through Fut8 and then regulates the macrophage migration process. LCA lectin blot was used to detect whether glycosylation occurred on α-1,6-linked N-acetylglucosamines. SNA, VVL, MALI, and PHA-L can also detect changes in other glycosylation forms. The results of IP experiments confirmed that Unc5b was modified by α-1,6 fucosylation after ox-LDL stimulation, as shown in Fig. 4A, B. However, the interactions between Unc5b and SNA, VVL, MALI, and PHA-L were not changed upon ox-LDL treatment (Fig. 4C, D). These results suggested that Unc5b was a fucosylated protein.

A previous study showed that N-glycan biosynthesis occurs in the endoplasmic reticulum (ER); N-glycans are produced as common oligomannosidic structures, and a number of N-glycan structures are produced via modification at specific glycosylation sites [33]. In the present study, we transfected HEK 293T cells with the pCMV-Fut8-mCherry plasmid as well as pCMV-Unc5b-GFP plasmid or pCMV-Unc5b^ko222,347^-GFP plasmid along with an ER marker to detect the integrity of Unc5b as well as its interaction with Fut8. As shown in Fig. 4E, Unc5b was strongly colocalized with Fut8 in the ER, as indicated by the white arrows. However, after knockout of Unc5b fucosylation sites such as asparagine 222/347, Unc5b was unable be fucosylated by Fut8 and was partially aggregated in the ER and could not localize to cell membranes, as shown in Fig. 4E. These results further suggested that fucosylation of Unc5b mediated by Fut8 mainly occurs in the ER. In addition, we found that down-regulation of Fut8 would lead to hypofucosylation of Unc5b, as shown in Fig. 4F. Together, these results suggested that the Unc5b protein was modified by α-1,6 fucosylation by interacting with Fut8.

### Unc5b regulates macrophage migration through the p-CDC42/p-PAK pathway

**Fig. 5:**
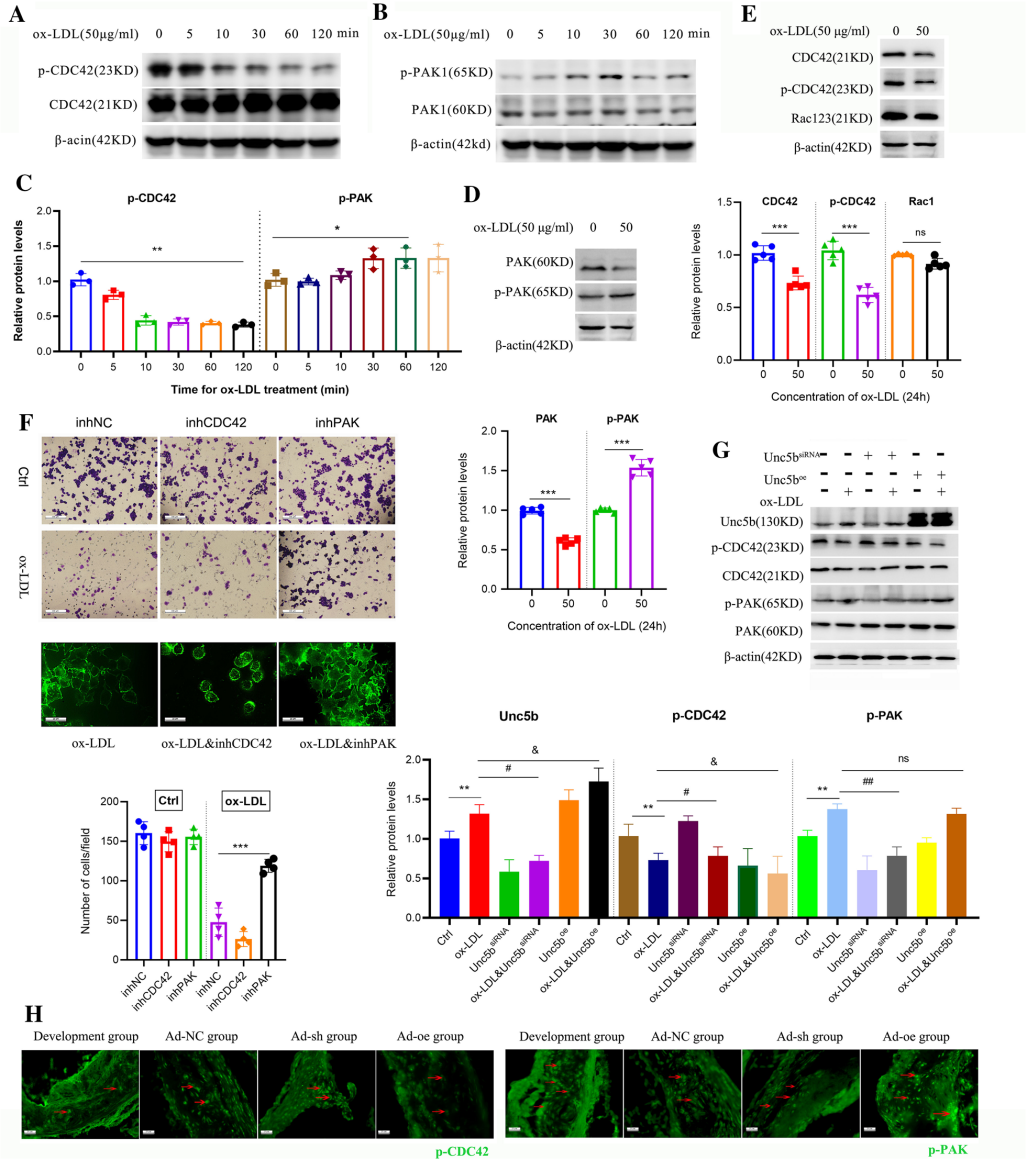
Unc5b affected macrophage migration through p-CDC42/p-PAK pathway. The expression of CDC42, p-CDC42 **A** as well as PAK, p-PAK **B** in Raw 264.7 cells after ox-LDL stimulation for 0-120 min were analyzed by Western blotting; **C** Quantification of p-CDC42 and p-PAK expression from Western blotting. Data are reported as the mean ± S.D. (n＞3, **, p< 0.01vs vehicle group, *p< 0.05 vs vehicle group, t-test). **D** CDC42, p-CDC42 and Rac123 protein levels in Raw 264.7 cells were analyzed by Western blotting. **E** Western blot analysis of PAK and p-PAK protein levels in Raw 264.7 cells. **F** Raw 264.7 cells were pretreated with CDC42 inhibitor or PAK inhibitor, and then the number of cells on the dark side was identified by Transwell assay upon ox-LDL (50 μg/ml) treatment. Also the effects of CDC42 inhibitor or PAK inhibitor on the formation of macrophage pseudopodium was detected by F-actin staining (bar=20 μm, n=4). **G** Raw 264.7 cells were pretreated with Unc5b^siRNA^ or Unc5b^oe^ combined with ox-LDL (50 μg/ml) stimulation, the Unc5b, CDC42 and PAK protein levels were determined by Western blotting. **H** Atherosclerotic aortic sinus were collected from ApoE^−/−^ mice that was fed a Western diet (Development group) for 16 weeks, and also injected with Ad-NC, Ad-sh or Ad-oe for another 4 weeks. The aortic arch was stained for p-CDC42 and p-PAK (green).  Data are reported as the mean ± S.D. (n=4, *, p< 0.05 ; ***, p< 0.001; t-test.)

Rho GTPases are regarded as modulators of the actin cytoskeleton and are involved in processes such as cell migration, cell polarity, and membrane trafficking. Specifically, Rac1/2/3, CDC42 and their serine/threonine protein kinase PAK are the most critical proteins involved in phagocytosis and cell migration [34]. Next, we examined whether Rho GTPases participate in the regulation of Unc5b-mediated macrophage migration. First, we treated Raw264.7 cells with ox-LDL for 120 min and then evaluated the protein levels of CDC42, p**-**CDC42, PAK, and p-PAK. Western blot analysis showed that the level of p-CDC42 was already decreased after 10 min and exhibited time dependency after ox-LDL stimulation. Additionally, ox-LDL increased the level of p-PAK by 2-fold at 60 min compared with that in the vehicle group without ox-LDL treatment (Fig. 5A, B, C). Furthermore, we evaluated the protein levels of CDC42, p**-**CDC42 and Rac1/2/3 after ox-LDL stimulation for 24 h. The results showed that ox-LDL also decreased the protein levels of CDC42 and p-CDC42 but had no effect on those of Rac1/2/3 (Fig. 5D). Similarly, the protein levels of PAK and p-PAK after ox-LDL treatment for 24 h were analyzed and are shown in Fig. 5E. The level of p-PAK was significantly increased along with the decrease in PAK, which preliminarily indicated that ox-LDL activated the p-PAK/p-CDC42 pathway.

To further verify the role of the p-PAK/p-CDC42 pathway, we pretreated Raw264.7 cells with inhibitors of CDC42 and PAK as described in the Materials and Methods section, and cells with ox-LDL treatment were regarded as control. We observed fewer migrated cells on the underside of the membrane and a slower migration speed, as well as a reversal of F-actin staining in the group treated with the CDC42 inhibitor compared with ox-LDL group. However, we also observed the opposite effects after treatment with the PAK inhibitor, which reversed the inhibition of macrophage migration induced by ox-LDL (Fig. 5F). To establish the relationship between Unc5b and activation of the p-PAK/p-CDC42 pathway, we used the Unc5b^siRNA^ and Unc5b^oe^ cell models to detect expression changes in CDC42 and PAK. The western blot results showed that Unc5b^oe^ cells displayed a decreased level of p-CDC42 and an increased level of p-PAK, while cells Unc5b^siRNA^ cells displayed the opposite pattern (Fig. 5G). Furthermore, we detected the activity of p-CDC42/p-PAK in the atherosclerotic aortic arch. As shown in Fig. 5H, p-CDC42 and p-PAK are marked with red arrows in atherosclerotic plaques. Mice treated with Ad-sh displayed lower levels of p-PAK and higher levels of p-CDC42 than those treated with Ad-oe compared with the Ad-NC group, which was consistent with the in vitro results. Collectively, these data demonstrated that Unc5b affected macrophage migration through a decrease in the p-CDC42 level and an increase in the p-PAK level upon ox-LDL treatment.

## Discussion

Researchers have found that plaques with foamy macrophage retention are one of the hallmarks of atherosclerotic damage [7, 35]. Ox-LDL has been shown to disrupt lipid organization and affect macrophage activation, cytokine production and foam cell formation. However, its impact on the regulation of macrophage retention remains unclear [36]. Our previous work showed that ox-LDL induced macrophage foaming and that Unc5b was strongly activated in foamy macrophages. Afterward, the migration ability of foam cells is eventually reduced in vitro [11]. Our present study further confirmed that hypofucosylation of Unc5b regulated by Fut8 was essential for the migration ability of foam cells both in vivo and in vitro and that the p-CDC42/p-PAK signaling pathway was the main participant in the regulatory process.

Regulated glycosylation of proteins is an important step in regulating protein folding, localization and function. Fut8 is defined as α-1,6-fucosyltransferase and is the only enzyme known to generate a-1,6-fucosylated structures on the core of N-glycans residue. Researcher have confirmed that Fut8 could alter some functional properties through the induction of fucosylation, which is also a characteristic of cardiovascular diseases [19, 22, 24]. Our previous studies showed that aberrant fucosylation, especially α-1,6 fucosylation catalyzed by Fut8, is associated with reduced cell migration and the accumulation of cholesterol-enriched foam cells in the early stage of atherosclerosis [26]. The present study further confirmed that Fut8 strongly participated in macrophage activation and migration in response to ox-LDL. Moreover, we found that Unc5b was fucosylated by Fut8, which mainly mediated macrophage retention during the progression of atherosclerosis.

As previous studies have shown, Unc5b was initially discovered in a screen of *C. elegans* for motor dysfunctions and is expressed in endothelial cells as well as macrophages in mice and humans [15, 16]. Recent studies have also demonstrated that Unc5b has important functions in vascular development, which is mainly associated with its dysregulation in macrophages [37]. However, the regulatory role of Unc5b in macrophage retention is unclear. In addition, Unc5b has been reported to be a glycoprotein, and whether its glycosylation levels are changed also remains unclear. The present study confirmed that Unc5b was mainly localized on the cell membrane and modified by Fut8 both in vivo and in vitro. Deletion of the glycosylation sites at asparagine 222 and 347 affects Unc5b localization and decreases its interaction with Fut8, thereby affecting the macrophage migration capacity.

Actin polymerization occurs under many conditions within cells, and the numbers of migrating cells and pseudopodia are considered the most well-characterized markers of actin polymerization [38]. The hallmark family members Rho, Ras-related C3 botulinum toxin substrate (Rac), and cell division control protein 42 homolog (CDC42) are important regulators that affect actin polymerization and cell migration [36, 39]. Our results showed that CDC42 could regulate pseudopod formation and affect the macrophage migration capacity, whereas Rac could not. Additionally, PAK is a mammalian Rac/CDC42-associated serine/threonine protein kinase. The biological function of PAK depends on N-terminal and C-terminal-mediated protein‒protein interactions and downstream substrate phosphorylation signals [33, 34]. Huang et al. found that knockdown of CDC42 inhibited the migration and proliferation of M2 macrophages [34]. Shiraishi et al. confirmed that CDC42 was activated during macrophage migration [33]. In the present study, we further confirmed that CDC42 and its regulator PAK were both inhibited, accompanied by inhibition of macrophage migration. We also found that the p-CDC42 level was decreased and the p-PAK level was increased during the macrophage retention process. Moreover, Unc5b regulated the migration process through activation of the p-PAK/p-CDC42 signaling pathway, which highlights the potential of this receptor as a therapeutic target for atherosclerosis.

However, there were some deficiencies in the present study. As reported, systemic administration of inducible adenoviruses is a relatively common method for establishing mouse models of gene overexpression or partial knockdown. Zhang et al. used systemic administration of Ad-Atg14 or Ad-LacZ to investigate the effect of ATG14 in macrophages on the development of atherosclerosis, similar to the method used in our present research [40]. However, systemically administered Unc5b adenoviruses could also infect vascular endothelial cells or exert other effects such as inducing cellular degradation in normal tissue, which may also influence the process of atherogenesis. Therefore, further research in mice with macrophage-specific Unc5b overexpression or knockout will be conducted and will provide more specific evidence to clarify the role of Unc5b in atherosclerosis.

## Conclusion

In conclusion, we demonstrated the negative role of Unc5b fucosylation in the migration of foam cells. Additionally, we clarified the mechanism of Unc5b in regulating macrophage-derived foam cells during the plaque regression process. This study provides a novel potential therapeutic target for atherosclerosis and diseases with inflammatory vascular injury.

## Materials and methods

### Chemicals and reagents

Ox-LDL was purchased from Yiyuanbiotech (YB-002, Guangzhou, China). The anti-CDC42 antibody, anti-Rac1/2/3 antibody, anti-PAK antibody, anti-pCDC42 antibody, anti-pPKA antibody, and Phalloidin-iFluor 488 were purchased from Abcam (Oxford, UK). The anti-Unc5b antibody was purchased from CST (Danvers, USA). The PAK inhibitor (FRAX486) and CDC42 inhibitor (ML141) were purchased from MCE (MCE, USA). Biotin-labeled LTL, biotin-labeled LCA, biotin-labeled AAL, biotin-labeled UEA1, biotin-labeled SNA, biotin-labeled MAL1, biotin-labeled VVL, and biotin-labeled PHA-L were purchased from Vector Laboratories (Peterborough, UK). ER-Tracker Blue‒White DPX was purchased from Yeasen Biotech Co., Ltd. (Shanghai, China). Oil Red O solution was purchased from Solarbio Science & Technology Co., Ltd. (Beijing, China). FBS was purchased from BI (Israel).

### Cell culture and treatment

Human HEK293A, human HEK293T and murine Raw264.7 cell lines were obtained from ATCC (Manassas, US). Cells were cultured in DMEM containing 10% FBS at 5% CO_2_ and 37 °C.

### Isolation of mouse peritoneal macrophages

Isolation of mouse peritoneal macrophages was performed according to a previous study [25]. Male C57BL/6J mice (8 weeks old) were purchased from the Animal Experimental Center of Chongqing Medical University. In brief, mice were intraperitoneally injected with 1 mL of 3% thioglycollate medium. Then, after 5–7 days, the mice were euthanized by CO_2_ inhalation, and 10 mL of PBS was injected into the abdominal cavity to wash out the macrophages, which were then used for further experiments.

### Atherosclerosis model establishment, treatment and analysis of atherosclerosis

Forty-eight male ApoE^−/−^ mice (6 weeks old) were purchased from the Animal Experimental Center of Chongqing Medical University. The male ApoE^−/−^ mice were randomly divided into six groups: the vehicle group (n = 8), in which ApoE^−/−^ mice were maintained on a normal diet for 12 weeks and were then euthanized by CO_2_ inhalation; the baseline group (n = 8), in which ApoE^−/−^ mice were maintained on a Western diet containing 21% fat and 0.15% cholesterol for 12 weeks (MD12015, Medscience Ltd., Jiangsu, China) and were then euthanized by CO_2_ inhalation; the development group (n = 8), in which ApoE^−/−^ mice were maintained on a Western diet for 16 weeks and were the euthanized by CO_2_ inhalation; the Ad-NC group (n = 8); the Ad-sh group (n = 8); and the Ad-oe group (n = 8). The mice in the latter three groups were maintained on a Western diet for 16 weeks. In addition, in mice in the Ad-NC group, Ad-sh group and Ad-oe group, the corresponding adenovirus (1 × 10^8^ to 1 × 10^10^ PFU) was injected into the caudal vein for 4 weeks; for more details, please refer to the adenovirus section.

At the end of the experiments, the mice were sacrificed by CO_2_ inhalation and perfused with PBS, after which the aortic arteries and liver were removed and either frozen in OCT compound or fixed with 4% paraformaldehyde (PFA) for immunohistochemistry. Oil Red O staining combined with H&E staining was used to determine the lipid content in the plaque area and pathological structure, and Image Pro-Plus 6.0 (Media Cybernetics, Rockville, MD, USA) software was used to evaluate the sizes of plaque areas with positive staining and the intimal thickness of the aortic valve in the different groups.

This study was carried out in accordance with the recommendations of the Chongqing Management Approach of Laboratory Animals (Chongqing government order no. 195). The protocol was approved by the Institutional Review Board of Chongqing Medical University (Reference Number: CQMU 2010-26).

### Adenoviral constructs, packaging and purification

The Unc5b overexpression and knockdown adenoviruses were partially constructed by using pHBAd-U6-CMV-Vector (Hanbio, Shanghai, China) and partially constructed by using pDC316-mCMV-EGFP-vector (Biowit, Shenzhen, China) according to the manufacturer’s instructions. The adenovirus was then packaged in HEK293A cells cultured in DMEM containing 10% FBS. When the cells were approximately 90% confluent, adenoviral particles were added for infection for 48 h, and visible regions of CPE were observed. After a CPE was observed over approximately 70% of the cells, the adenovirus stock was harvested and stored at -80 °C. The adenovirus was purified using an Adenovirus Purification Kit (Sartorius, UK).

### Real-time PCR

The details of the staining procedure were as previously described [25, 26]. Total RNA was extracted from cells using an RNAeasy™ Animal RNA Isolation Kit with Spin Column (Beyotime Biotechnology, Beijing, China, R0026). Total RNA was subjected to RT with the PrimeScriptTM RT Reagent Kit (Takara, Dalian, China). Real-time PCR was performed using an iCycleriQ real-time detection system with SYBR Premix Ex TaqTM II.

### Western blot analysis

Western blot analysis was performed according to a previous paper [27]. Briefly, cells were treated with Cell Lysis Buffer for Western and IP (Beyotime Biotechnology, Beijing, China, P0013), which contained PMSF (Beyotime Biotechnology, Beijing, China, ST506). The protein concentrations in the samples were determined with a BCA protein assay kit. Then, proteins were separated via SDS–PAGE and transferred onto PVDF membranes. The membranes were probed separately with the primary antibodies and HRP-conjugated secondary antibodies.

### Immunoprecipitation (IP) assays

IP was performed in accordance with a previous study [28]. Briefly, RAW264.7 cells were treated with cell lysis buffer, and the protein concentrations were determined by a BCA protein assay kit. Next, 1 mg of protein was immunoprecipitated with 6 µL of the anti-Unc5b antibody in cell lysis buffer for the IP assay and incubated at 4 °C for 12 h. Then, protein A + G agarose beads (Beyotime Biotechnology, Beijing, China) were added to the mixture and incubated at 4 °C for 6 h. The beads were collected and centrifuged at 3000 × g for 5 min at 4 °C, and 20% 5× loading buffer was subsequently added. Then, the beads were heated at 95 ℃ for 10 min and centrifuged at 12,000 ×g for 10 min. After that, the precipitated proteins were subjected to western blot analysis.

### Immunofluorescence staining

Raw264.7 cells or tissue sections were deparaffinized and stained with antibodies specific for Unc5b, Fut8 or CD68 followed by fluorescence-labeled secondary antibodies. Nuclei were counterstained with 4′,6-diamidino-2-phenylindole (DAPI). Additionally, F-actin was stained using phalloidin-iFluor 488. The tissue sections or cells were visualized using a Nikon Eclipse 80i microscope with NIS Elements software.

### Small interfering RNA (siRNA) transfection

Raw264.7 cells were passaged in a 6-well plate at 10^6^ cells per well. After incubation overnight, two Eppendorf tubes—one containing 100 pmol siRNA in 50 µL of Opti-MEM (Thermo Fisher Scientific) and the other containing 8 µl of Lipofectamine™ RNAi MAX in 50 µL of Opti-MEM—were incubated for 5 min at room temperature, mixed together and incubated for another 20 min at RT. The siRNA/Lipofectamine mixture was added dropwise into the wells. The sequences of the siRNA targeting Unc5b were as follows: 5′-GGA CGC UAC UUG ACU CCA ATT − 3′ (sense) and 5′- GGA CUA CUU GAC UCC GCA ATT − 3′ (antisense).

### Plasmid transfection

The transfection reagent Lipofectamine 3000 was purchased from Invitrogen (Invitrogen Co., Carlsbad, CA, USA). Cells at approximately 50–70% confluence were transfected with the recombinant pcDNA3.1-Unc5b plasmid, pcDNA3.1-Fut8 plasmid and pcDNA3.1 backbone plasmid according to the manual. Six hours later, the transfection medium was replaced with complete DMEM. Twenty-four hours later, 50 µg/mL ox-LDL was added to the cells.

### Transwell assay

The Transwell assay was performed according to a previous study with some modifications [25, 31]. Briefly, Raw264.7 cells were placed in the upper chamber, and 50 mg/mL ox-LDL was added and incubated for 24 h. Then, serum-free medium was added to the upper chamber, and DMEM containing 10% FBS was added to the lower chamber and incubated for another 24 h. Subsequently, cells on the underside of the membrane were fixed with 4% PFA for 10 min and stained with 0.1% crystal violet for 10 min.

### Wound healing assay

The wound healing assay was performed according to a previous study [31]. Briefly, Raw264.7 cells were treated with 50 mg/mL ox-LDL for 24 h, and a micropipette tip (10 mL white tip) was used to scratch the cell layers. Subsequently, the wounds were gently rinsed with PBS, and the cells were then incubated in serum-free medium for 24 h.

### Statistical analysis

The data are presented as the mean ± SD from at least triplicate determinations. The data were statistically analyzed using one-way ANOVA with proper post hoc tests or by two-tailed Student’s t test. Statistical analysis was performed using GraphPad Prism 8.0.1. A value of *p* < 0.05 was considered significant.

## Supplementary Information

**Additional file 1: Figure S1.** Feed intake and weight changes in the body, heart,liver and spleen. (A) Feed intakeoutcome and Weight outcome. n=6, Data are reported as the mean ± S.D. (B) Theweights of the heart, liver and spleen in the atherosclerosis model asindicated in the figure recorded. Data are reported as the mean ± S.D. (n=6, ***,p< 0.0001 versus vehicle l group, t-test.).

**Additional file 2: Figure S2.** Immunofluorescence stainging of normal IgG in aortic sinusAtheroscleroticaortic sinus were collected from ApoE−/− mice in Development group, Ad-NC, Ad-sh andAd-oe group. The aortic sinus was stained for Unc5b (red), Fut8 (green), CD68(blue) and their with plaque morphology (merge). (n=3).

## Data Availability

All data generated or analyzed during this study are included in this published article. Data and material will be made available on reasonable request.

## Abbreviations

AS: Atherosclerosis
CVD: Cardiovascular disease
Unc5b: Uncoordinated-5 homolog B receptor
Fut8: Fucosyltransferase 8
ApoE −/−: Apolipoprotein E-deficient
ox-LDL: Oxidized low-density lipoprotein
HEK 293T cells: Human embryonic kidney cells
Raw264.7 cells: Mouse macrophages
CDC42: Cell division control protein 42 homolog
PAK: p21-activated kinase
Rac: Ras-related C3 botulinum toxin substrate
