# Supplementary figures and images for: Hypofucosylation of Unc5b regulated by Fut8 enhances macrophage emigration and prevents atherosclerosis
Source: Cell Biosci. 2023 Jan 20;13:13. doi: 10.1186/s13578-023-00959-y (PMC9854080; doi:10.1186/s13578-023-00959-y)

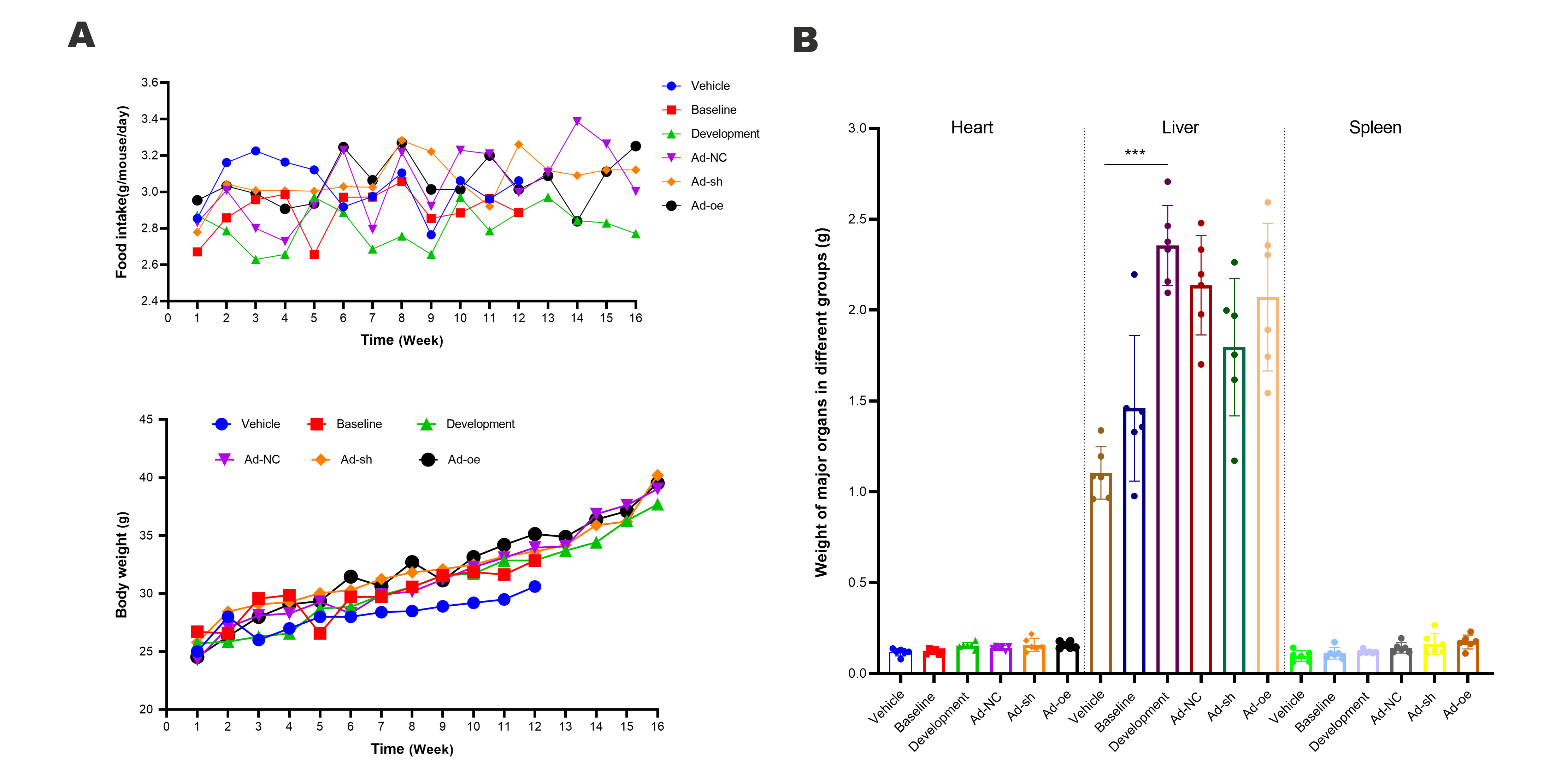

Supplement: Supplementary file 1 — Additional file 1: Figure S1. Feed intake and weight changes in the body, heart,liver and spleen. (A) Feed intakeoutcome and Weight outcome. n=6, Data are reported as the mean ± S.D. (B) Theweights of the heart, liver and spleen in the atherosclerosis model asindicated in the figure recorded. Data are reported as the mean ± S.D. (n=6, ***,p< 0.0001 versus vehicle l group, t-test.). [file 13578_2023_959_MOESM1_ESM.jpg]

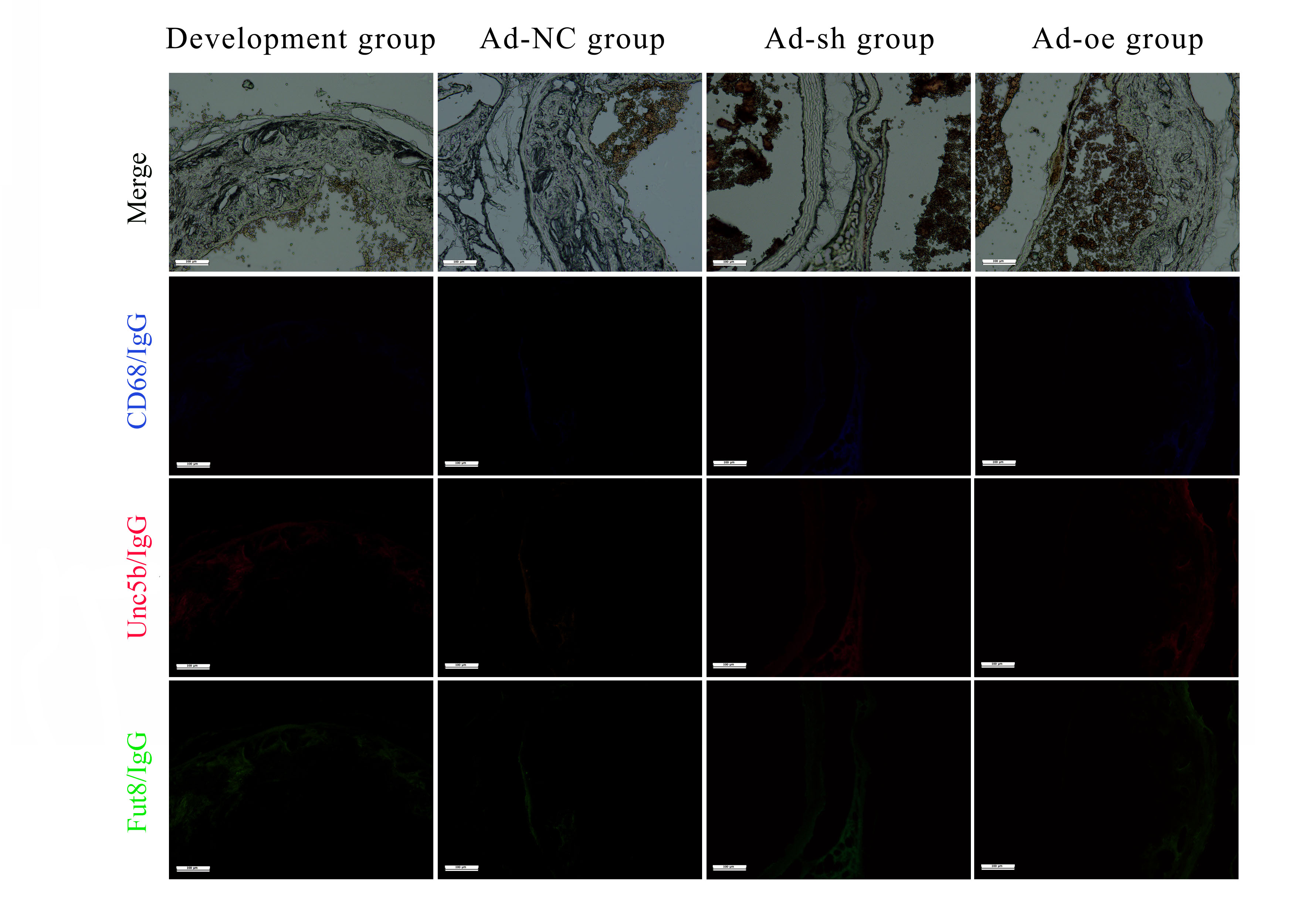

Supplement: Supplementary file 2 — Additional file 2: Figure S2. Immunofluorescence stainging of normal IgG in aortic sinusAtheroscleroticaortic sinus were collected from ApoE−/− mice in Development group, Ad-NC, Ad-sh andAd-oe group. The aortic sinus was stained for Unc5b (red), Fut8 (green), CD68(blue) and their with plaque morphology (merge). (n=3). [file 13578_2023_959_MOESM2_ESM.jpg]
